# Erythema nodosum and the risk of tuberculosis in a high incidence setting

**DOI:** 10.3402/ijch.v75.32666

**Published:** 2016-10-25

**Authors:** Karen Bjorn-Mortensen, Karin Ladefoged, Jacob Simonsen, Sascha W. Michelsen, Hans Christian F. Sørensen, Anders Koch, Troels Lillebaek, Aase Bengaard Andersen, Bolette Soborg

**Affiliations:** 1 Department of Epidemiology Research, Statens Serum Institut, Copenhagen, Denmark; 2 International Reference Laboratory of Mycobacteriology, Statens Serum Institut, Copenhagen, Denmark; 3 Greenland's Center of Health Research, Nuuk, Greenland; 4 Institute of Clinical Medicine, University of Southern Denmark, Odense, Denmark; 5 Department of Internal Medicine, Queen Ingrid's Hospital, Nuuk, Greenland; 6 Tasiilaq Hospital, Tasiilaq, Greenland; 7 Department of Infectious Diseases, Copenhagen University Hospital, Copenhagen, Denmark

**Keywords:** Erythema Nodosum, interferon gamma release assay, mycobacterium tuberculosis, Tuberculosis, Greenland

## Abstract

**Objective:**

This study estimates the erythema nodosum (EN) incidence in a tuberculosis (TB) endemic setting and evaluates the likelihood of a subsequent TB diagnosis among individuals with *Mycobacterium tuberculosis* infection (MTI) with or without EN.

**Design:**

We estimated EN incidence rates (IRs) in East Greenland in 2010–2011 and conducted a cohort study following all individuals who tested positive for MTI from 1 January 2010 until 31 December 2012. A personal identifier allowed individual follow-up in the mandatory TB register. MTI was defined by a positive interferon-gamma release assay. TB incidence rate ratios (IRRs) among participants with or without EN were estimated with the Cox proportional hazard model.

**Results:**

We identified 38 EN cases corresponding to an IR of 500/100,000 inhabitants/year. All cases were among individuals with MTI. The EN IR was 11.79 (95% CI 5.73–24.27) times higher for BCG-unvaccinated compared with BCG-vaccinated individuals. The TB IRR was 25 (95% CI 11–60) within 1 month of EN compared to individuals without EN.

**Conclusion:**

This study documents a high EN incidence in a TB endemic region. EN occurred only in individuals with MTI, and predominantly among BCG-unvaccinated individuals. EN was significantly associated with a TB diagnosis within 1 month of diagnosis.

Erythema nodosum (EN), a septal panniculitis presenting with tender, red nodules bilaterally on the extremities without ulceration and scarring, is a well-known immunologic reaction to numerous different stimuli such as infections, drugs and malignant diseases (1,2). The proportion of EN cases associated with *Mycobacterium tuberculosis* (*Mtb*) infection (MTI) was highly debated in the early 1900s (3–6). While some authors reported approximately 20–30% of EN cases to be associated with tuberculosis (TB) (3,5), others reported a potential association in 100% of EN cases (6,7). Later, longitudinal studies demonstrated how EN incidence decreased in a population in parallel with a decreasing TB incidence (8–10). Even though EN associated with MTI is still common in some areas (11–14), most EN cases are associated with other diseases, and MTI is considered a rare cause (1,2,15,16).

East Greenland is a region with an extremely high TB incidence and MTI prevalence (17,18). In 2005, a screening of schoolchildren in the region found approximately 7% of the children to have MTI (19). However, 7 years later, following a TB outbreak, more than 40% of children were infected, indicating high levels of *Mtb* transmission between 2005 and 2012 (17–19). Likewise, as documented in our recently published study based on more than 4,000 interferon-gamma release assays (IGRAs), the MTI population prevalence in East Greenland increased dramatically in this period (18).

During a population-wide MTI settlement screening in East Greenland in 2010, clinicians noted a high EN prevalence. Almost simultaneously, the clinicians at the local hospital began registering all incident EN cases from the region. Within 2 years, almost 40 cases of the characteristic skin eruptions were registered.

The aim of this study was to estimate the yearly EN incidence in East Greenland from 2010 to 2011 and to evaluate the likelihood of being diagnosed with TB among individuals with MTI with or without EN.

## Methods and materials

### Setting

Greenland is a self-governed country within the Kingdom of Denmark. Approximately 3,500 of Greenland's 56,000 inhabitants populate East Greenland. The East Greenlandic population is mainly Inuit, and the majority live in the town of Tasiilaq and five nearby settlements (18,20). The main hospital in the region is situated in Tasiilaq. Access to health care and treatment is free. At birth, all Greenlanders receive a personal identification number through the Civil Registration System (CRS), which is subsequently used in all public registers. The CRS number provides a unique possibility to combine data across public registers as well as obtaining information on age, gender, past and present places of residency and place of birth.

Since 1955, neonatal BCG vaccination has been part of the Greenlandic childhood vaccination programme except for a nationwide discontinuation of the programme from 1991 through 1996 (17,21). Therefore, individuals born from 1991 through 1996 were considered unvaccinated. This assumption was validated in a previous study (17). For BCG-vaccinated birth cohorts, the vaccination coverage was more than 90% (22).

### Case definitions

An EN case was defined as a clinical case of EN recorded by a doctor or a nurse in one of the two registers: (a) the 2010–2011 local hospital register and (b) the register from the cross-sectional settlement screening in 2010.

TB cases were identified from the mandatory Greenlandic TB notification register maintained by the National Board of Health. The Greenlandic TB case definitions follow WHO definitions (23,24). Hence, microbiologically confirmed TB cases were defined by at least one sample positive for acid-fast bacilli at microscopy, *Mtb* at culture or *Mtb* DNA at polymerase chain reaction.

A case of MTI was defined by a positive IGRA (Quantiferon TB Gold-in tube test, Cellestis/Qiagen) used as recommended by the manufacturer. IGRA results from the East Greenlandic population were obtained either from the Central Laboratory at Queen Ingrid's Hospital in Nuuk or from Statens Serum Institut (SSI) in Copenhagen. IGRAs from the Central Laboratory were from routine TB diagnostics, contact tracing, schoolchildren screenings (n=1,707) and population screenings (n=1,626), while IGRA from the SSI were obtained from previous studies (n=1,444) (17–19). In total, IGRAs from 2,791 East Greenlanders were included in the study; of whom, 990 individuals had at least one positive IGRA from 1 January 2005 through before 31 December 2012.

### Study design

We estimated the yearly crude EN incidence rate (IR) in 2010 and 2011 in the entire East Greenlandic population (N_pop_ per 1 January 2011=3,494) and among East Greenlanders with MTI (n=990). Furthermore, we conducted a cohort study to assess whether individuals with MTI and EN had a higher risk of TB as compared with individuals with MTI without EN. East Greenlandic inhabitants with MTI were included from the date of first positive IGRA if this came after 1 January 2010 and before an EN or TB diagnosis. Participants were followed until the date of TB notification, emigration, death or end of follow-up on 31 December 2012.

### Statistical analysis

The crude EN IRs and 95% Wald confidence intervals (CIs) were calculated from all registered EN cases diagnosed in 2010 and 2011 as number per 100,000 inhabitants per year. The crude EN IRs were compared for BCG-unvaccinated individuals (born 1991–1996) and BCG-vaccinated individuals (born before 1991 or after 1996) and compared for women and men.

In the cohort study, EN was considered as a time-dependent covariate because included individuals could change status from *having no EN* to *having EN* during the study period. TB incidence rate ratios (IRRs) with 95% CI were estimated using the Cox proportional hazard model with age as the underlying time scale. Estimates were adjusted for gender and year of birth in 5-year intervals. SAS statistical software version 9.4 was used for all analyses.

## Results

Overall, 38 cases of EN were registered, of which 34 (89%) could be validated in medical records. Among these 34 cases, 30 (88%) were described as typical examples of EN with red, tender, often painful nodular eruptions of the skin, while 4 were simply designated as “EN.” The 31 cases (91%) with detailed descriptions of the location of the skin eruptions all reported location on the lower legs. For 17 cases (50%), the location was further described as being on the anterior side of the legs, and none were described on the posterior side. Three cases (8%) had additional eruptions on the arms, and 21 cases (66%) were reported with systemic symptoms such as fever, night sweats, cough and weight loss at the time of EN diagnosis.

The 38 EN cases corresponded to an overall crude IR of 544 EN cases per 100,000 inhabitants per year (Table I), with 18 cases registered in 2010 and 20 cases registered in 2011. The majority of EN cases were among women (58%). Median age at the time of EN diagnosis was 16 years (interquartile range: 14–19 years).

**Table I:** Yearly crude incidence rates (IRs) with 95% confidence intervals (CIs) of registered erythema nodosum (EN) cases in East Greenland 2010–2011 in the total population and among individuals with *M. tuberculosis* infection (MTI)

		East Greenlandic population		East Greenlanders with MTI	
			
	EN cases (%)	N_pop_ (% of N_pop_)	IR (95% CI)	N (% of N_pop_)	IR (95% CI)
Entire population					
Total	38 (100)	3,494 (100)	544 (396–747)	990 (28)	1,919 (1,396–2,638)
Gender					
Women	23 (61)	1,678 (48)	685 (455–1,031)	472 (28)	2,436 (1,619–3,666)
Men	15 (39)	1,816 (52)	413 (248–685)	518 (29)	1,448 (872–2,401)
Year of birth					
Before 1991 (BCG-vaccinated)	6 (16)	2,120 (61)	142 (64–314)	660 (31)	455 (204–1,012)
1991–1996 (BCG-unvaccinated)	28 (74)	350 (10)	4,000 (2,762–5,793)	190 (54)	7,368 (5,088–10,672)
After 1996 (BCG-vaccinated)	4 (10)	1,024 (29)	195 (73–520)	140 (14)	1,429 (536–3,806)

IRs calculated as the number of EN cases per 100,000 inhabitants per year. EN was diagnosed in daily clinical routine 2010–2011 or at a TB screening in 2010. N_pop_ is the number of inhabitants per 1 January 2011. MTI was defined by a positive interferon-gamma release assay before the end of 2012.

All EN cases were diagnosed among the 990 individuals who had tested positive for MTI. Among these, the yearly EN IR was 1.68 (95% CI 0.88–3.22) times higher among women as compared with men and 11.79 (95% CI 5.73–24.27) times higher among BCG-unvaccinated individuals.

### EN and risk of TB

**Fig. 1:**
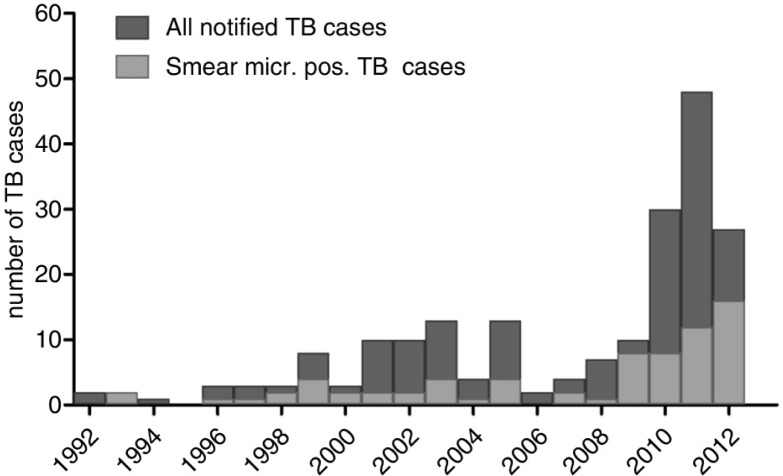
The number of notified tuberculosis (TB) cases (n=203) from 1992 to 2012 among the 990 East Greenlanders with *Mycobacterium tuberculosis* infection (MTI).

Of the total 990 individuals with MTI, 21% (n=203) were notified with TB. Of these, 48% (n=98) were notified in 1992–2009, while 52% (n=105) were notified in 2010–2012 (Fig. 1). Eighty-seven per cent (n=176) were pulmonary. Of the 952 individuals without EN, 19% (n=181) were notified with TB, of which 65% (n=118) were laboratory confirmed; compared to 22 (58%) notifications among the 38 individuals with EN, 59% (n=13) were confirmed.

Only 739 individuals with MTI were included in the cohort study, since the remainder had their first positive IGRA test before 1 January 2010 or after EN or TB diagnosis. Of these 739 individuals, 67% (n=494) were born before 1991, 17% (n=129) were born between 1991 and 1996 and 16% (n=116) were born after 1996. During follow-up, 46 individuals developed TB without prior EN as compared with 20 individuals with EN (Table II). The majority of individuals with EN were diagnosed with TB within the first month after EN diagnosis (n=16). Thus, among individuals with MTI, the IRR of TB within 1 month after EN was 25 (95% CI 11–60) as compared with TB without prior EN. This finding was robust for both genders and all birth cohorts. When only including confirmed TB cases, our estimates did not change to any greater extent.

**Table II:** Incidence rate ratios (IRRs) of tuberculosis (TB) among 739 East Greenlanders with a first positive interferon-gamma release assay (IGRA) after 1 January 2010 and before erythema nodosum (EN) or TB diagnosis

	All notified TB cases			Confirmed TB cases		
		
	Person years	Cases	IRR (95% CI)	Person years	Cases	IRR (95% CI)
All						
No prior EN	1,032	46	1 (ref)	1,086	27	1 (ref)
0–30 days after EN	3.6	16	25 (11–60)	4.9	9	13 (4.4–37)
> 30 days after EN	39	4	0.8 (0.3–2.5)	55	3	0.7 (0.2–2.4)
Women						
No prior EN	498	19	1 (ref)	516	13	1 (ref)
0–30 days after EN	2.2	9	44 (13–153)	3.1	5	13 (2.9–60)
> 30 days after EN	23	3	1.2 (0.3–4.1)	32	3	1.3 (0.3–5.0)
Men						
No prior EN	534	27	1 (ref)	571	14	1 (ref)
0–30 days after EN	1.4	7	15 (4.3–49)	1.7	4	13 (2.7–59)
> 30 days after EN	16	1	0.4 (0.1–3.6)	23	0	NA
Born before 1991 (BCG-vaccinated)						
No prior EN	743	27	1 (ref)	779	16	1 (ref)
0–30 days after EN	0.7	1	7.7 (0.6–96)	1,0	0	NA
> 30 days after EN	7.7	1	2.6 (0.3–22)	12	1	3,0 (0.3–26)
1991–1996 (BCG-unvaccinated)						
No prior EN	113	19	1 (ref)	128	11	1 (ref)
0–30 days after EN	2.3	15	37 (15–90)	3.2	9	40 (12–135)
> 30 days after EN	22	2	0.5 (0.1–2.2)	34	1	0.3 (0.04–2.4)
After 1996 (BCG-vaccinated)						
No prior EN	176	0	1 (ref)	179	0	1 (ref)
0–30 days after EN	0.6	0	NA	0.6	0	NA
> 30 days after EN	9.9	1	NA	9.9	1	NA

Individuals without a diagnosis of EN compared to individuals diagnosed with TB less than 30 days after EN or more than 30 days after EN. IRRs were estimated with Cox proportional hazard model with age as the underlying time scale. Estimates were adjusted for gender and year of birth in 5-year intervals.

## Discussion

This study documents a high EN incidence in East Greenland, a TB high incidence region. From 2010 to 2011, the crude yearly IR was approximately 500 per 100,000 inhabitants. All cases were among individuals with MTI with an 11.8 times higher IR for BCG-unvaccinated individuals compared to BCG-vaccinated individuals. The risk of developing TB among individuals with MTI was significantly and dramatically increased within the first month after EN.

EN is believed to appear in immunocompetent individuals with a strong immunologic response. EN cases often present strong reactions to the tuberculin skin test (TST), which represents delayed hypersensitivity reactions (25–27). Consistent with this, a recent study demonstrated an excess cytokine response (increased production of INFγ) to *Mtb* antigens in EN patients (25). Additionally, blood from EN patients was shown to exhibit an enhanced ability to restrict mycobacterial growth in vitro.

In this study, BCG-unvaccinated individuals had the highest risk of developing EN when infected with *Mtb*. For the entire East Greenlandic population, the crude IR of EN among these individuals was 20–30 times higher than that among BCG-vaccinated individuals. The higher proportion of individuals with MTI among unvaccinated individuals (Table I) could explain most of this effect. However, when we included only individuals with MTI in the analysis, BCG-unvaccinated individuals still had an almost 12-fold increased EN IR, suggesting that the immunologic response to newly acquired MTI might be different for BCG-unvaccinated individuals as compared to BCG-vaccinated individuals. The increased risk of EN in BCG-unvaccinated East Greenlandic individuals did not imply an enhanced ability to restrict mycobacterial growth, since it has been documented that the same individuals had a higher risk of MTI and TB (17). However, it does appear that the immune response to newly acquired MTI differs according to BCG vaccination status. Although this is an interesting observation, it is beyond the scope of this paper to explore this topic further.

Several studies suggest that EN be considered as a symptom of underlying TB rather than exclusively a response to newly acquired MTI (14,28,29). In this study, approximately 50% of individuals with MTI and EN were subsequently diagnosed with TB; the majority were diagnosed within 1 month after EN with a 25-fold increased risk during this time period as compared to individuals with MTI and no EN. Thus, in this study, the risk of TB was very high among individuals with MTI and EN, suggesting EN as either a strong predictor of TB or an early symptom of TB. Either way, EN can be used to identify individuals with MTI with the highest likelihood of a subsequent TB diagnosis, who should be examined carefully and followed closely. This was already proposed in 1938 by Wallgreen who suggested that EN be considered as a “favourable syndrome” which aids early diagnosis and treatment of pulmonary TB (28). Our findings, in accordance with earlier studies, led to an interesting discussion on whether individuals with MTI and EN should be treated for TB or with preventive monotherapy. WHO guidelines stress the need to exclude TB before prescribing preventive monotherapy in order to avoid the development of drug resistance (30). In accordance with our findings, it seems highly relevant to consider screening for MTI and TB in individuals presenting with EN. However, in hard to reach populations with limited possibility of continuous monitoring for TB symptoms, it might be wise to initiate TB treatment in patients with MTI and EN.

The major strength of this study is the population-based design, the well-maintained registers and the unique possibility of data linkage on a personal level. However, some potential biases can be discussed. We assumed that all individuals born before or after the period 1991–1996 were BCG-vaccinated at birth. When including the entire East Greenlandic population, not regarding place of birth, some individuals in the assumed BCG-vaccinated cohorts might be unvaccinated. However, as immigration to East Greenland is low, we consider potential misclassification of BCG-vaccination status to be of minor importance. No biopsies were available for histopathological validation of EN diagnoses. However, other causes such as HIV infection are almost non-existent in the region; malnutrition is rare and type 2 diabetes is unlikely to be relevant in our EN cases, who had a median age of 16 (31). In addition, almost all EN cases were validated in medical records, and for a majority of cases, the description of EN lesions was very detailed. Thus, we believe EN diagnoses to be correct. The EN IR in this study represents a minimum incidence only, since only individuals with severe or painful eruptions were likely to have consulted the health care system. In addition, EN cases were not systematically registered in the remote town of Ittoqqortoormiit, which would potentially result in misclassification of EN cases as non-EN cases. On the contrary, clinicians in East Greenland could have been sensitized to make an EN diagnosis after the recognition of a TB outbreak in the region. However, the data in the hospital register were collected prospectively as symptomatic individuals presented themselves at the local hospital and not by any systematic screening effort. Likewise, the clinician treating individuals with EN could have been more prone to do a closer TB examination and be more inclined to diagnose TB, which might lead to observer bias. However, when only including confirmed TB cases in our analysis, the association between EN and subsequent TB was the same as when we included all notified TB cases. Due to the small number of cases in our study, stratifying the findings would have been limited. However, both methods showed a significant increase in IRR of TB within 1 month of EN which reassures us of the close relationship between EN and a subsequent TB diagnosis. Hence, we chose to adjust the findings rather than stratify.

This study reminds us that in a TB high incidence setting, EN among individuals with MTI is common, and MTI should not be considered a rare cause of EN as described in recent literature (1,2,15,16). Many publications on EN aetiology are from countries where TB is rare and where MTI is not prevalent (32–35), which might explain the difference in EN incidence and aetiology in different settings. Indeed, in older reports, EN is often described as a clear indication of MTI (6). EN was believed to appear approximately at the same time as tuberculin reactivity and is therefore considered as a symptom of newly acquired infection (7). In East Greenland, this fits well with the timeframe, since the unusually high number of EN cases was reported approximately at the same time as the TB incidence started to increase and new *Mtb* transmission was occurring (18).

In conclusion, this study documents a high incidence of EN in a TB high incidence setting. EN occurred only among individuals with MTI and predominantly among BCG-unvaccinated individuals. Since the diagnosis of EN was significantly associated with the diagnosis of TB within the first month, this study strongly suggests that individuals with MTI presenting with EN have an increased likelihood of a subsequent TB diagnosis.
